# Serum leptin levels in relation to circulating cytokines, chemokines, adhesion molecules and angiogenic factors in normal pregnancy and preeclampsia

**DOI:** 10.1186/1477-7827-9-124

**Published:** 2011-09-09

**Authors:** Attila Molvarec, András Szarka, Szilvia Walentin, Gabriella Bekő, István Karádi, Zoltán Prohászka, János Rigó Jr

**Affiliations:** 1 First Department of Obstetrics and Gynecology, Semmelweis University, Budapest, Hungary; 2 Central Laboratory, Kútvölgyi Clinical Center, Semmelweis University, Budapest, Hungary; 3 Department of Laboratory Medicine, Semmelweis University, Budapest, Hungary; 4 Third Department of Internal Medicine, Semmelweis University, Budapest, Hungary; 5 Research Group of Inflammation Biology and Immunogenomics, Hungarian Academy of Sciences, Budapest, Hungary

## Abstract

**Objective:**

In this study, we determined circulating levels of C-reactive protein, several cytokines, chemokines, adhesion molecules and angiogenic factors along with those of leptin in healthy non-pregnant and pregnant women and preeclamptic patients, and investigated whether serum leptin levels were related to the clinical characteristics and measured laboratory parameters of the study participants.

**Methods:**

Sixty preeclamptic patients, 60 healthy pregnant women and 59 healthy non-pregnant women were involved in this case-control study. Levels of leptin and transforming growth factor (TGF)-beta1 in maternal sera were assessed by ELISA. Serum levels of interleukin (IL)-1beta, IL-1 receptor antagonist (IL-1ra), IL-2, IL-4, IL-6, IL-8, IL-10, IL-12p40, IL-12p70, IL-18, interferon (IFN)-gamma, tumor necrosis factor (TNF)-alpha, interferon-gamma-inducible protein (IP)-10, monocyte chemotactic protein (MCP)-1, intercellular adhesion molecule (ICAM)-1 and vascular cell adhesion molecule (VCAM)-1 were determined by multiplex suspension array. Serum C-reactive protein (CRP) concentrations were measured by an autoanalyzer. Serum total soluble fms-like tyrosine kinase-1 (sFlt-1) and biologically active placental growth factor (PlGF) levels were determined by electrochemiluminescence immunoassay. For statistical analyses, non-parametric methods were applied.

**Results:**

There were significant differences in most of the measured laboratory parameters among the three study groups except for serum IL-1beta and TGF-beta1 levels. Serum leptin levels were significantly higher in preeclamptic patients and healthy pregnant women than in healthy non-pregnant women. Additionally, preeclamptic patients had significantly higher leptin levels as compared to healthy pregnant women. Serum leptin levels were independently associated with BMI in healthy non-pregnant women. In healthy pregnant women, both BMI and serum CRP concentrations showed significant positive linear association with leptin levels. There were significant positive correlations between serum leptin concentrations of healthy pregnant women and systolic blood pressure, as well as serum levels of IP-10, while their serum leptin levels correlated inversely with fetal birth weight. In preeclamptic patients, a significant positive correlation was observed between serum concentrations of leptin and IP-10. Furthermore, elevated serum leptin level and sFlt-1/PlGF ratio had an additive (joint) effect in the risk of preeclampsia, as shown by the substantially higher odds ratios of their combination than of either alone.

**Conclusions:**

Simultaneous measurement of leptin with several inflammatory molecules and angiogenic factors in this study enabled us to investigate their relationship, which can help to understand the role of circulating leptin in normal pregnancy and preeclampsia.

## Background

Preeclampsia, characterized by hypertension and proteinuria developing after the 20^th ^week of gestation in a previously normotensive woman, is a severe complication of human pregnancy with a worldwide incidence of 2-10%. It is one of the leading causes of maternal, as well as perinatal morbidity and mortality, even in developed countries. Despite extensive research, the etiology and pathogenesis of preeclampsia are not completely understood. There is an increasing body of evidence that an exaggerated maternal systemic inflammatory response to pregnancy with activation of both the innate and the adaptive arms of the immune system plays a central role in the pathogenesis of the disease [1,2]. The excessive production of pro-inflammatory cytokines, chemokines and adhesion molecules may trigger a generalized endothelial dysfunction characteristic of the maternal syndrome of preeclampsia [3]. In addition, an imbalance between angiogenic and anti-angiogenic factors has also been implicated in the development of this multifactorial disorder [4-6].

Leptin is a peptide hormone of 16 kDa molecular weight comprising 167 amino acids. The major source of leptin is the adipose tissue, but it can also be produced by other organs, including the placenta [7]. This anti-obesity hormone decreases food intake and increases energy expenditure, thereby reducing body weight and adiposity [8]. It also modulates glucose metabolism by increasing insulin sensitivity [9] and activates the sympathetic nervous system [10,11]. Furthermore, leptin has been implicated in the control of the reproductive function, including embryonic development and implantation [12]. Leptin can also be considered as a pro-inflammatory cytokine that belongs to the type I cytokine superfamily and has structural similarity with interleukin-6 [13]. Increasing evidence suggests that leptin is involved in the regulation of innate and adaptive immune responses and inflammation [14]. Circulating leptin levels are significantly higher in pregnant than in non-pregnant women [7,15], and there is a further increase in complicated pregnancies, such as gestational diabetes mellitus, preeclampsia and intrauterine growth restriction [16-20].

In the present study, we determined serum leptin levels in a large number of healthy non-pregnant and pregnant women and preeclamptic patients. We also measured circulating levels of C-reactive protein and several cytokines, chemokines, adhesion molecules and angiogenic factors in a comprehensive manner, and investigated whether serum leptin levels were related to the clinical characteristics and measured laboratory parameters of the study participants.

## Methods

### Study patients

Our study was designed as a case-control study. Sixty preeclamptic patients, 60 healthy pregnant women with uncomplicated pregnancies and 59 healthy non-pregnant women were involved in the study. The study participants were enrolled in the First Department of Obstetrics and Gynecology and in the Department of Obstetrics and Gynecology of Kútvölgyi Clinical Center, at the Semmelweis University, Budapest, Hungary. All women were Caucasian and resided in the same geographic area in Hungary. The preeclamptic patients and healthy pregnant women were matched on the basis of maternal age and gestational age at blood draw, and they were selected accordingly from the previously reported groups of 93 preeclamptic patients and 176 healthy pregnant women [21,22]. Exclusion criteria were multifetal gestation, chronic hypertension, diabetes mellitus, autoimmune disease, angiopathy, renal disorder, maternal or fetal infection and fetal congenital anomaly. None of the pregnant women were in active labor, and none had rupture of membranes. The healthy non-pregnant women were consecutively selected in the early follicular phase of their menstrual cycle (between cycle days 3 and 5), and none of them received hormonal contraception.

Preeclampsia was defined by increased blood pressure (≥140 mmHg systolic or ≥90 mmHg diastolic on ≥2 occasions at least 6 hours apart) that occurred after the 20^th ^week of gestation in a woman with previously normal blood pressure, accompanied by proteinuria (≥0.3 g/24 h or ≥1 + on dipstick in the absence of urinary tract infection). Blood pressure returned to normal by the 12^th ^postpartum week in each preeclamptic study patient. Preeclampsia was regarded as severe if any of the following criteria was present: blood pressure≥160 mmHg systolic or ≥110 mmHg diastolic, or proteinuria≥5 g/24 h (or ≥3 + on dipstick). Pregnant women with eclampsia or HELLP syndrome (hemolysis, elevated liver enzymes, and low platelet count) were not enrolled in this study. Early onset of preeclampsia was defined as onset of the disease before the 34^th ^week of gestation (between the 20^th ^and 33^rd ^completed gestational weeks). Intrauterine growth restriction (IUGR) was diagnosed if the fetal birth weight was below the 10^th ^percentile for gestational age and gender, based on Hungarian birth weight percentiles [23].

The study protocol was approved by the Regional and Institutional Committee of Science and Research Ethics of the Semmelweis University, and written informed consent was obtained from each patient. The study was conducted in accordance with the Declaration of Helsinki.

### Biological samples

Fasting blood samples were taken from an antecubital vein into plain tubes, and then centrifuged at room temperature with a relative centrifugal force of 3000 *g *for 10 minutes. The aliquots of serum were stored at -80°C until the analyses.

### Laboratory methods

Levels of leptin and transforming growth factor (TGF)-β1 in maternal sera were assessed by ELISA (DRG International, Mountainside, New Jersey, USA, Cat. No. EIA-2395 and EIA-1864, respectively). Serum levels of interleukin (IL)-1β, IL-1 receptor antagonist (IL-1ra), IL-2, IL-4, IL-6, IL-8, IL-10, IL-12p40, IL-12p70, IL-18, interferon (IFN)-γ, tumor necrosis factor (TNF)-α, interferon-γ-inducible protein (IP)-10, monocyte chemotactic protein (MCP)-1, intercellular adhesion molecule (ICAM)-1 and vascular cell adhesion molecule (VCAM)-1 were determined by multiplex suspension array on a Bio-Plex 200 analyzer (Bio-Rad Laboratories, Hercules, California, USA). We diluted the serum 1:4 in sample diluent. The samples were measured at low Photomultiplier Tube (PMT) setting according to the instructions provided in the assay manual. The assays were performed using the Bio-Plex filter plates and manual vacuum manifold. We applied the Bio-Plex Manager™software. A 10-point extended broad range standard curve was used in order to maximize sensitivity for samples that have very low levels of analytes. Any high end saturation points were removed from the standard curves for determining sample concentrations. Serum C-reactive protein (CRP) concentrations were measured by an autoanalyzer (Cobas Integra 800, Roche, Mannheim, Germany) using the manufacturer's kit. Serum total soluble fms-like tyrosine kinase-1 (sFlt-1) and biologically active placental growth factor (PlGF) levels were determined by electrochemiluminescence immunoassay (Elecsys, Roche, Mannheim, Germany, Cat. No. 05109523 and 05144671, respectively) on a Cobas e 411 analyzer (Roche, Mannheim, Germany).

### Statistical analysis

The normality of continuous variables was assessed using the Shapiro-Wilk's *W*-test. As the continuous variables were not normally distributed, non-parametric statistical methods were used. To compare continuous variables between two groups, the Mann-Whitney *U*-test was applied, whereas to compare them among multiple groups, the Kruskal-Wallis analysis of variance by ranks test was performed. Multiple comparisons of mean ranks for all groups were carried out as post-hoc tests. The Fisher exact and Pearson χ^2 ^tests were used to compare categorical variables between groups. The Spearman rank order correlation was applied to calculate correlation coefficients. Analysis of covariance (ANCOVA) and multiple linear regression analyses were undertaken, as a non-parametric method, with logarithmically transformed values of serum leptin concentrations. Odds ratios (OR) with 95% confidence intervals (CI) were calculated by logistic regression. For all statistical analyses, p < 0.05 was considered statistically significant.

In the article, data are reported as median (interquartile range) for continuous variables and as number (percentage) for categorical variables.

## Results

### Patient characteristics

The clinical characteristics of the study participants are described in Table 1. There was no statistically significant difference in terms of age among the study groups. Furthermore, no significant differences were observed in gestational age at blood collection and the percentage of primiparas between preeclamptic patients and healthy pregnant women. However, all of the other clinical features presented in Table 1, including smoking status, differed significantly among our study groups. Fetal growth restriction was absent in healthy pregnant women, whereas the frequency of this condition was 18.3% in the preeclamptic group. Doppler abnormalities were observed in 7 fetuses of preeclamptic mothers (11.7%). Twenty-one women (35.0%) had severe preeclampsia and 5 patients (8.3%) experienced early onset of the disease.

**Table 1:** Clinical characteristics of healthy non-pregnant and pregnant women and preeclamptic patients

	Healthy non-pregnant women (n = 59)	Healthy pregnant women (n = 60)	Preeclamptic patients (n = 60)
Age (years)	28 (23-35)	30 (28-32)	29 (26-32)
Pre-pregnancy BMI (kg/m^2^)	n.a.	21.0 (19.5-22.6)	25.5 (21.6-28.1)^d^
BMI at blood draw (kg/m^2^)	20.8 (19.6-22.9)	25.8 (24.3-27.9)^b^	29.9 (26.9-33.3)^b, d^
Smokers	14 (23.7%)	0 (0%)^b^	3 (5.0%)^a^
Primiparas	n.a.	37 (61.7%)	38 (63.3%)
Systolic blood pressure at blood draw (mmHg)	115 (110-120)	110 (107-120)	162 (155-180)^b, d^
Diastolic blood pressure at blood draw (mmHg)	80 (70-80)	70 (60-80)^b^	100 (97-110)^b, d^
Gestational age at blood draw (weeks)	n.a.	36 (36-37)	37 (36-39)
Gestational age at delivery (weeks)	n.a.	39 (38-40)	38 (37-39)^d^
Fetal birth weight (grams)	n.a.	3450 (3150-3700)	3125 (2450-3475)^d^
Fetal growth restriction	n.a.	0 (0%)	11 (18.3%)^d^

Data are presented as median (interquartile range) for continuous variables and as number (percentage) for categorical variables

n.a.: not applicable; BMI: body mass index

^a ^p < 0.05 versus healthy non-pregnant women

^b ^p < 0.001 versus healthy non-pregnant women

^c ^p < 0.05 preeclamptic patients versus healthy pregnant women

^d ^p < 0.001 preeclamptic patients versus healthy pregnant women

### Laboratory parameters

**Figure 1:**
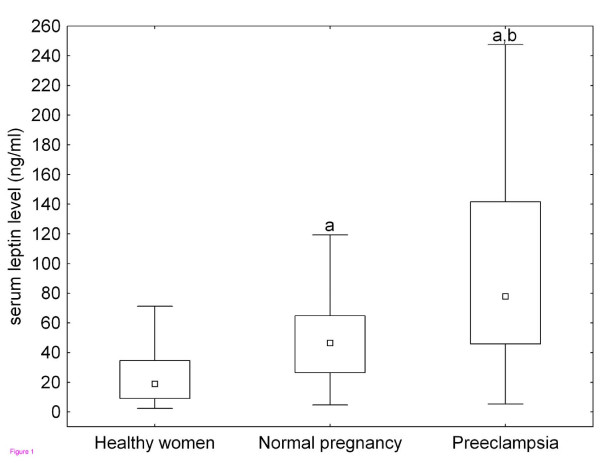
**Serum leptin levels of healthy non-pregnant and pregnant women and preeclamptic patients**. Middle point: median; Box: interquartile range (25-75 percentile); Whisker: range (excluding outliers). ^a ^p < 0.001 versus healthy non-pregnant women; ^b ^p < 0.001 preeclamptic patients versus healthy pregnant women.

**Figure 2:**
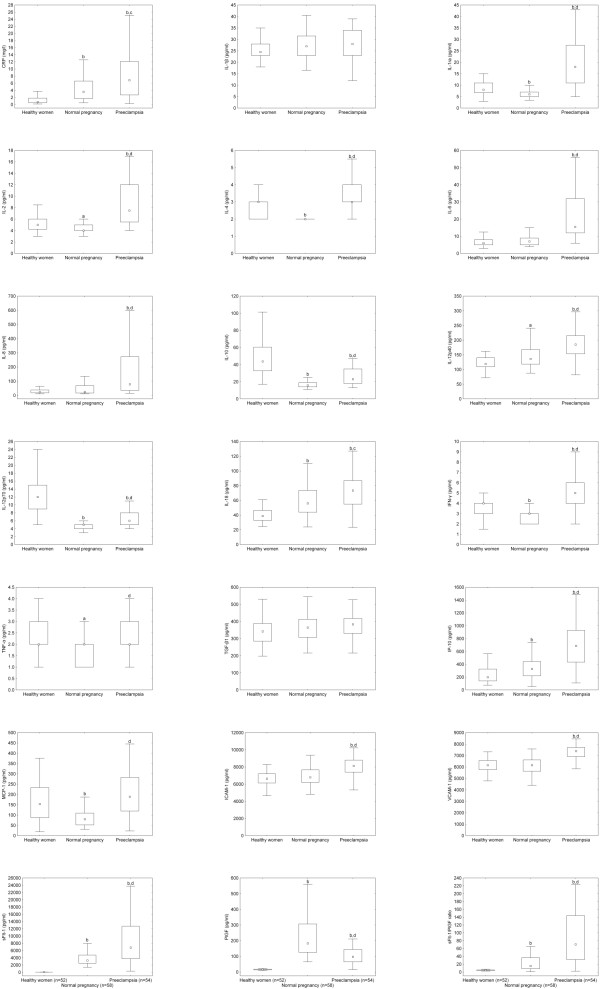
**Serum levels of C-reactive protein, cytokines, chemokines, adhesion molecules and angiogenic factors in healthy non-pregnant and pregnant women and preeclamptic patients**. Middle point: median; Box: interquartile range (25-75 percentile); Whisker: range (excluding outliers). CRP: C-reactive protein; IL: interleukin; IL-1ra: IL-1 receptor antagonist; IFN: interferon; TNF: tumor necrosis factor; TGF: transforming growth factor; IP: interferon-γ-inducible protein; MCP: monocyte chemotactic protein; ICAM: intercellular adhesion molecule; VCAM: vascular cell adhesion molecule; sFlt: soluble fms-like tyrosine kinase; PlGF: placental growth factor. ^a ^p < 0.05 versus healthy non-pregnant women; ^b ^p < 0.001 versus healthy non-pregnant women; ^c ^p < 0.05 preeclamptic patients versus healthy pregnant women; ^d ^p < 0.001 preeclamptic patients versus healthy pregnant women.

The laboratory parameters of the study subjects are displayed in Figure 1 and 2. As can be seen in the figures, there were significant differences in most of the measured laboratory parameters among the three study groups except for serum IL-1β and TGF-β1 levels. As shown in Figure 1, serum leptin levels were significantly higher in preeclamptic patients and healthy pregnant women than in healthy non-pregnant women. Furthermore, preeclamptic patients had significantly higher leptin levels as compared to healthy pregnant women. The differences remained significant even after adjustment for BMI at blood draw in ANCOVA.

In the group of preeclamptic patients, no statistically significant differences were found in serum leptin concentrations between patients with mild and severe preeclampsia, between patients with late and early onset of the disease, or between preeclamptic patients with and without fetal growth restriction (data not shown).

### Relationship of serum leptin levels of the study subjects to their clinical characteristics and laboratory parameters

We also investigated whether serum leptin levels of the study participants were related to their clinical features and laboratory parameters by calculating the Spearman rank order correlation coefficients (continuous variables) or by the Mann-Whitney *U*-test (categorical variables). In healthy non-pregnant women, serum leptin concentrations correlated significantly with body mass index (BMI) and serum CRP levels (Spearman R = 0.59, p < 0.001 and 0.39, p < 0.05, respectively). However, in multiple linear regression analysis, only the association between leptin levels and BMI remained significant (standardized regression coefficient (β) = 0.53, p < 0.001). In the group of healthy pregnant women, we found statistically significant positive correlations between serum leptin concentrations and BMI (R = 0.49, p < 0.001), systolic blood pressure (R = 0.38, p < 0.05), as well as serum levels of CRP (R = 0.46, p < 0.05), IL-6 (R = 0.35, p < 0.05) and IP-10 (R = 0.31, p < 0.05). In a multiple linear regression model involving BMI, serum CRP and IL-6 levels as independent variables, both BMI and serum CRP concentrations showed significant positive linear association with leptin levels in healthy pregnant women (β = 0.43 and 0.45, respectively, p < 0.05 for both). There was a significant inverse correlation between serum leptin concentrations of healthy pregnant women and fetal birth weight (R = -0.36, p < 0.05), even after adjustment for gestational age at delivery in multiple linear regression analysis (β = -0.51, p < 0.001). In the preeclamptic group, a significant positive correlation was observed between serum levels of leptin and IP-10 (R = 0.36, p < 0.05). There was no other relationship between serum leptin concentrations of the study subjects and their clinical features and measured laboratory parameters in either study group.

**Figure 3:**
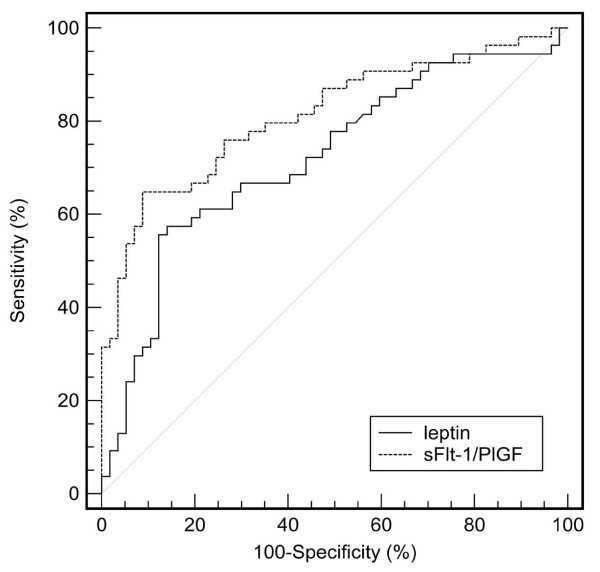
**Receiver Operating Characteristic (ROC) curves**. Serum leptin levels (leptin, continuous line) and soluble fms-like tyrosine kinase-1 to placental growth factor ratio (sFlt-1/PlGF ratio, dashed line) to discriminate between preeclamptic patients and healthy pregnant women.

Using the Receiver Operating Characteristic (ROC) curve analysis (Figure 3), we determined cut-off values for serum leptin concentration (> 74.3 ng/ml, sensitivity: 57.6%, specificity: 84.7%; area under curve (AUC) with 95% CI: 0.72 (0.63-0.80)) and sFlt-1/PlGF ratio (> 31.2, sensitivity: 75.9%, specificity: 74.1%; AUC (95% CI): 0.81 (0.73-0.88)) to discriminate preeclamptic patients from healthy pregnant women. In a multiple logistic regression model involving both variables, elevated serum leptin level and sFlt-1/PlGF ratio were found to be independent predictors of preeclampsia (odds ratios with 95% confidence intervals: 9.16 (3.16-26.5) and 8.89 (3.34-23.7), respectively, p < 0.001 for both; after adjustment for BMI at blood draw: 5.03 (1.48-17.1), p < 0.05 and 11.3 (3.24-39.2), p < 0.001, respectively). Women with elevated serum leptin level and sFlt-1/PlGF ratio had substantially higher odds for having preeclampsia than those with elevated sFlt-1/PlGF ratio or serum leptin concentration alone (OR (95% CI): 80.5 (14.4-450), p < 0.001 versus 9.0 (2.80-29.0), p < 0.001 and 9.33 (2.27-38.4), p < 0.05, respectively), even after adjustment for BMI at blood collection in multiple logistic regression analyses (adjusted OR (95% CI): 65.9 (9.47-459), p < 0.001 versus 6.91 (1.55-30.7), p < 0.05 and 1.42 (0.15-13.0), p > 0.05, respectively).

## Discussion

In this study, we measured circulating levels of C-reactive protein, several cytokines, chemokines, adhesion molecules and angiogenic factors along with those of leptin in healthy non-pregnant and pregnant women and preeclamptic patients. According to our findings, serum leptin levels were independently associated with BMI in healthy non-pregnant women. In healthy pregnant women, both BMI and serum CRP concentrations showed significant positive linear association with leptin levels. There were significant positive correlations between serum leptin concentrations of healthy pregnant women and systolic blood pressure, as well as serum levels of IP-10, while their serum leptin levels correlated inversely with fetal birth weight. In preeclamptic patients, a significant positive correlation was observed between serum concentrations of leptin and IP-10. Furthermore, the combination of elevated serum leptin level and sFlt-1/PlGF ratio was found to be additive for the risk of preeclampsia.

Leptin was first introduced as an adipocyte-derived signal of energy metabolism [24]. Indeed, the independent association of serum leptin levels with BMI in our healthy non-pregnant and pregnant women indicates that adipose tissue is a major source of circulating leptin. Later on, the placenta has been demonstrated to be an alternative source of leptin in the maternal circulation [7,25]. Maternal serum leptin levels have been reported to increase during the course of a normal pregnancy with a peak at around 20-30 weeks of gestation and to decrease rapidly after birth [26-28]. In addition to BMI, serum levels of CRP were also directly related to those of leptin in our healthy pregnant group (unlike in non-pregnant women [29]), suggesting that systemic inflammation characteristic of the third trimester of normal pregnancy might contribute to the increase in circulating leptin levels. In fact, inflammatory stimuli, including pro-inflammatory cytokines, can induce the expression of leptin and increase its serum level [30-32]. Therefore, the elevation of maternal circulating leptin levels in normal pregnancy could reflect - at least in part - an increase in adipocyte/trophoblast leptin synthesis mediated by inflammatory stimuli. On the contrary, serum leptin levels were found to increase independently from BMI and CRP in our preeclamptic group, as shown by the lack of correlations with these variables. Interestingly, placental expression of leptin has been observed to be raised in preeclampsia by several research groups, including ours [17,33-37]. An increasing body of evidence suggests that placental insufficiency and resultant placental hypoxia might be responsible for augmented placental production of leptin in this pregnancy-specific disorder, leading to its elevated concentration in the maternal circulation [17,38-41].

Leptin is a pleiotropic molecule, which has also been implicated in the regulation of innate and adaptive immune responses by several ways [14,42]. This molecule alters the balance of T cell-derived cytokines in favour of a Th1-type response [43,44]. Leptin can also induce pro-inflammatory cytokine production of monocytes/macrophages [45,46]. It is noteworthy that the maternal syndrome of preeclampsia is characterized by a predominant Th1-type immune response with elevated amounts of pro-inflammatory molecules in the maternal circulation [3,47,48]. However, in this study, we did not find any association between serum levels of leptin and pro-inflammatory cytokines in preeclampsia, which might be explained - at least partly - by the fact that the latter (especially TNF-α and IL-1β) have a very short half- life in the maternal circulation. Leptin may also be involved in blood pressure regulation through its central effect on sympathetic activity [10,11,49]. Accordingly, there was a significant positive correlation between serum leptin concentrations and systolic blood pressure in our healthy pregnant women. Nevertheless, the lack of relationship between leptin levels and blood pressure values in our preeclamptic group indicates that the process responsible for blood pressure elevation in this multifactorial disease is more complex and involves the interplay of numerous factors in addition to the sympathetic action of leptin.

An intriguing finding of our study is that increased serum levels of leptin were related to those of interferon-γ-inducible protein (IP)-10 both in normal pregnancy and preeclampsia, as shown by the significant correlations between these variables. This is consistent with the *in vitro *observation that leptin selectively induces the expression and secretion of IP-10 in human monocytic cells [50]. IP-10 (CXCL10) has pro-inflammatory and anti-angiogenic properties, and this chemokine has been proposed to be a potential link between inflammation and anti-angiogenesis in preeclampsia [51]. In our previous study, IP-10 showed the strongest association with markers of endothelial dysfunction, as well as with renal and liver function parameters in healthy pregnant women and preeclamptic patients [3]. Therefore, it is tempting to speculate that IP-10 might play a connecting role between elevated circulating leptin levels and the generalized endothelial dysfunction characteristic of preeclampsia. On the other hand, leptin can promote angiogenesis at least partly through the up-regulation of vascular endothelial growth factor expression in endothelial cells [52,53]. Nevertheless, increased levels of anti-angiogenic factors such as sFlt-1 in the maternal circulation in normal pregnancy and preeclampsia might blunt this beneficial direct effect of leptin on angiogenesis.

Another remarkable observation of this study is that serum leptin levels correlated inversely with fetal birth weight in the group of healthy pregnant women. In previous studies, elevated circulating leptin levels have been found in mothers with fetal growth restriction [18,19]. This relationship might reflect the state of inflammation and chronic stress in IUGR mothers. As reduced uteroplacental blood flow plays an important role in the pathogenesis of IUGR, resultant placental hypoxia might also account for the observed increase in maternal circulating leptin levels. Indeed, leptin expression was up-regulated in placental tissues of IUGR neonates [54]. Furthermore, maternal first trimester plasma leptin levels negatively correlated with birth weight among normotensive and preeclamptic women [55]. Interestingly, we have recently detected an inverse correlation between IP-10 levels and fetal birth weight in healthy pregnant women, which raises the possibility that maternal leptin levels could influence fetal growth in normal pregnancy - at least in part - through the inhibitory effect of IP-10 on placental angiogenesis. Nevertheless, preeclampsia may disrupt the association of maternal leptin levels with fetal growth, as shown by the lack of correlation with birth weight in our preeclamptic group. On the contrary, a recent meta-analysis demonstrated that cord blood leptin concentrations of small-for-gestational-age newborns are significantly lower than those of appropriate-for-gestational-age newborns [56]. Additionally, fetal circulating leptin levels positively correlate with birth weight in normal pregnancy [15,57]. In fact, leptin seems to play a crucial role in fetal growth [58], and it might be a potential link between poor fetal growth and the subsequent development of chronic diseases in later life [59].

Accumulating data indicate a central role of circulating angiogenic factors and their antagonists in the pathogenesis of preeclampsia [4-6]. In this study, we also measured serum sFlt-1 and PlGF concentrations in normal pregnancy and preeclampsia by electrochemiluminescence immunoassay. However, increased sFlt-1 and decreased PlGF levels were not related to serum leptin concentrations in women with preeclampsia, suggesting that alterations in angiogenic cytokine profile and circulating levels of leptin are independent mechanisms in the pathogenesis of this multifactorial disorder. Instead, elevated serum leptin level and sFlt-1/PlGF ratio had an additive (joint) effect in the risk of preeclampsia, as shown by the substantially higher odds ratios of their combination than of either alone.

In our study, the similar circulating leptin concentrations of preeclamptic patients regardless of the severity, the time of onset of the disease or the presence of fetal growth restriction might be explained by the multifactorial etiology of preeclampsia. Several genetic, behavioural and environmental factors need to interact to produce the complete picture of this pregnancy-specific disorder. Our research group reported various genetic (including leptin receptor gene polymorphisms) and soluble factors that were associated with the severity or complications of preeclampsia [60-63]. Nevertheless, it is also possible that the relatively small sample size of this study prevented to detect an effect in the subgroup analyses.

Simultaneous measurement of leptin with C-reactive protein, several cytokines, chemokines, adhesion molecules and angiogenic factors in this study enabled us to investigate their relationship, which can help to understand the role of circulating leptin in normal pregnancy and preeclampsia. Elevated leptin concentration in the first trimester of pregnancy appears to be associated with an increased risk of developing preeclampsia later in gestation [55,64,65]. Given the case-control design of our study, we could not confirm this association. Further longitudinal studies are required to clarify the predictive value of circulating leptin in preeclampsia.

## Conclusions

Elevated serum leptin concentrations were associated with the mass of adipose tissue, systemic inflammation and systolic blood pressure, as well as negatively with birth weight in the third trimester of normal pregnancy. In addition, increased serum levels of leptin were related to those of interferon-γ-inducible protein (IP)-10 both in normal pregnancy and preeclampsia, suggesting that circulating leptin might contribute to the development of endothelial dysfunction and poor fetal growth through the anti-angiogenic effect of IP-10. Moreover, the combination of elevated serum leptin level and sFlt-1/PlGF ratio was found to be additive for the risk of preeclampsia.

## Competing interests

The authors declare that they have no competing interests.

## Authors' contributions

AM conceived of the study, participated in its design and coordination, performed statistical analyses and drafted the manuscript. ASZ collected data and helped to draft the manuscript. SZW determined circulating angiogenic factors. GB carried out multiplex suspension array measurements. IK participated in the coordination of the study. ZP measured serum leptin levels. JR participated in the design and coordination of the study. All authors read and approved the final manuscript.
